# The causal relationship between gut microbiota and biliary tract cancer: comprehensive bidirectional Mendelian randomization analysis

**DOI:** 10.3389/fcimb.2024.1308742

**Published:** 2024-03-15

**Authors:** Kui Wang, Suijian Wang, Xianzheng Qin, Yifei Chen, Yuhua Chen, Jiawei Wang, Yao Zhang, Qiang Guo, Chunhua Zhou, Duowu Zou

**Affiliations:** ^1^ Department of Gastroenterology, Ruijin Hospital, School of Medicine, Shanghai Jiao Tong University, Shanghai, China; ^2^ Department of Gastroenterology, The Affiliated Hospital of Kunming University of Science and Technology, The First People’s Hospital of Yunnan Province, Kunming, China; ^3^ Department of Endocrinology, The First Affiliated Hospital, School of Medicine, Shantou University, Shantou, China; ^4^ The First Clinical Medical College, Lanzhou University, Lanzhou, Gansu, China; ^5^ Department of Critical Care Medicine, Jieyang Third People’s Hospital, Jieyang, Guangdong, China

**Keywords:** genome-wide association study, comprehensive bidirectional mendelian randomization, gut microbiota, biliary tract cancer, probiotics

## Abstract

**Background:**

Growing evidence has shown that gut microbiome composition is associated with Biliary tract cancer (BTC), but the causality remains unknown. This study aimed to explore the causal relationship between gut microbiota and BTC, conduct an appraisal of the gut microbiome’s utility in facilitating the early diagnosis of BTC.

**Methods:**

We acquired the summary data for Genome-wide Association Studies (GWAS) pertaining to BTC (418 cases and 159,201 controls) from the Biobank Japan (BBJ) database. Additionally, the GWAS summary data relevant to gut microbiota (N = 18,340) were sourced from the MiBioGen consortium. The primary methodology employed for the analysis consisted of Inverse Variance Weighting (IVW). Evaluations for sensitivity were carried out through the utilization of multiple statistical techniques, encompassing Cochrane’s Q test, the MR-Egger intercept evaluation, the global test of MR-PRESSO, and a leave-one-out methodological analysis. Ultimately, a reverse Mendelian Randomization analysis was conducted to assess the potential for reciprocal causality.

**Results:**

The outcomes derived from IVW substantiated that the presence of *Family Streptococcaceae* (OR = 0.44, *P* = 0.034), *Family Veillonellaceae* (OR = 0.46, *P* = 0.018), and *Genus Dorea* (OR = 0.29, *P* = 0.041) exerted a protective influence against BTC. Conversely, *Class Lentisphaeria* (OR = 2.21, *P* = 0.017), *Genus Lachnospiraceae FCS020 Group* (OR = 2.30, *P* = 0.013), and *Order Victivallales* (OR = 2.21, *P* = 0.017) were associated with an adverse impact. To assess any reverse causal effect, we used BTC as the exposure and the gut microbiota as the outcome, and this analysis revealed associations between BTC and five different types of gut microbiota. The sensitivity analysis disclosed an absence of empirical indicators for either heterogeneity or pleiotropy.

**Conclusion:**

This investigation represents the inaugural identification of indicative data supporting either beneficial or detrimental causal relationships between gut microbiota and the risk of BTC, as determined through the utilization of MR methodologies. These outcomes could hold significance for the formulation of individualized therapeutic strategies aimed at BTC prevention and survival enhancement.

## Introduction

Gallbladder cancer (GBC) and cholangiocarcinomas (CCAs) are cumulatively categorized under the umbrella term of biliary tract cancers (BTCs) (Benson et al., 2023). GBC is recognized as the principal malignancy impacting the biliary tract, characterized by an exceedingly poor prognosis, particularly in advanced stages. This is largely due to its aggressive invasion and the limited availability of efficacious treatment options (Roa et al., 2022). CCA is characterized as a highly lethal and heterogeneous primary liver cancer that originates from the biliary epithelium (Tomlinson et al., 2023; Ilyas et al., 2023). The incidence of BTC is on an upward trajectory globally, btc continues to constitute a significant global health concern. Projected mortality data pertaining to oncological conditions in the United States indicate that by the year 2040, hepatic and intrahepatic bile duct neoplasms are anticipated to overtake colorectal cancer, ascending to become the third leading etiology of cancer-associated fatalities (Rahib et al., 2021). However, the etiological factors contributing to CCA remain inadequately elucidated (Banales et al., 2020; Clements et al., 2020; Ouyang et al., 2021; Sung et al., 2021). The obstacles of screening and early detection persist, largely due to the infrequent manifestation of distinctive symptoms in patients (European Liver Research Association (2023); Harding et al., 2023; Kelley et al., 2023). The prompt diagnosis and categorization of BTC at its incipient stage is of critical importance to enhance the probability of therapeutic success. BTC may manifest as a consequence of the cumulative accrual of both genetic and epigenetic modifications. This pathogenesis is potentially modulated by a multitude of factors, including host immune responses, dietary habits, environmental elements, and microbial interactions (Nakamura et al., 2015; Kamisawa et al., 2017; Clements et al., 2020; Choi et al., 2022; Kendre et al., 2023; Jansson et al., 2023). The term ‘microbiome’ denotes the aggregate of genomic material stemming from microorganisms residing within a specific ecological niche (Sender et al., 2016). These microorganisms are essential in a range of host functions, including the modulation of immune responses, providing defense against pathogenic microbes, and managing metabolic regulatory processes (Honda and Littman, 2016; Brown et al., 2019).

The gastrointestinal tract and the liver share a profound anatomical and physiological interrelation, often referred to as the “gut-liver axis.” This axis governs not merely hepatic pathophysiological processes but also influences intrahepatic and systemic immune dynamics (Bubnov et al., 2019; Bubnov et al., 2017; Bubnov et al., 2015). Consequently, the gastrointestinal microbiota plays a pivotal role in regulating antineoplastic immune responses (Kudela et al., 2021). The intestinal barrier functions as the initial line of defense, a disrupted configuration of the intestinal microbiome, termed “dysbiosis,” has been linked to compromised integrity of the intestinal barrier (Peterson and Artis, 2014). The enhancement of our understanding of the microbiome’s role is substantially driven by advancements in high-throughput DNA sequencing and the refinement of computational techniques. These technological developments enable a more sophisticated examination of the microbiome’s intricacies (Wang et al., 2021).Emerging empirical data increasingly suggest that perturbations in the gut-liver axis may be instrumental in the etiopathogenesis of a myriad of hepatic disorders, including BTC (Scheufele et al., 2017; Ma et al., 2018; Schramm, 2018; Tripathi et al., 2018; Molinero et al., 2019; Wang et al., 2022). A recent study by Ma et al. confirmed that Helicobacter Species infection was associated with an increased risk of BTC (Gros et al., 2023). Zhou et al. observed a notably elevated prevalence of Helicobacter infections among individuals diagnosed with BTC, in contrast to those presenting with benign biliary conditions (Zhou et al., 2013). In the research conducted by Murphy and associates, they determined that there was a correlation between seropositivity to H. pylori proteins and an augmented likelihood of BTC onset (Murphy et al., 2014). The most efficacious approach to enhancing health outcomes appears to be through proactive prevention measures. Probiotics, supported by a robust body of evidence, demonstrate considerable potential in this preventative capacity. This presents significant prospects for the formulation of holistic strategies utilizing prebiotics for the promotion of healthful diets and the management and prophylaxis of BTC (Liu et al., 2022). Previous scholarly inquiries have indicated the potential of intestinal microbiota to act as an emergent biological indicator for the prognostic assessment and preventive measures concerning BTC (Plieskatt et al., 2013; Chng et al., 2016; Jia et al., 2020; Chen et al., 2021; Zhang Q. et al., 2021; Zhang T. et al., 2021; Mao et al., 2021; Abril et al., 2022; Binda et al., 2022; Ito et al., 2022; Okuda et al., 2022; Wheatley et al., 2022; Chai et al., 2023; Elvevi et al., 2023).

Nonetheless, the correlation between gut microbiota and BTC is susceptible to modulation by environmental variables, lifestyle choices, and additional confounding elements in observational investigations. Such conditions circumscribe the ability to draw causal inferences linking gut microbiota to BTC. The relationship between host genetic factors and the gut microbiome in the context of BTC necessitates more comprehensive exploration. Mendelian Randomization (MR), a prevalent analytical technique employed for investigating causal linkages between exposure variables and resultant outcomes, has been utilized to probe prospective causal affiliations between gut microbiota and a diverse array of medical conditions (Liu et al., 2023; Xi et al., 2023; Min et al., 2023; He et al., 2023; Li et al., 2023; Luo et al., 2023b). Consequently, we endeavored to ascertain the causal linkage between these variables at the genetic level through the application of MR techniques. Furthermore, we sought to identify potential microbial biomarkers conducive to disease prevention and amelioration in individuals diagnosed with BTC.

In the present study, we hypothesized that the incidence of BTC may be higher in patients with microbiota dysbiosis due to the fact that microbiota dysbiosis induces immune disruption through the gut-hepatic axis. Our objective is to attain personalized forecasting and prophylaxis of BTC by identifying specific microbiota in individual fecal samples that possess a causative association with BTC. This endeavor aims to furnish a robust evidence-based medicinal foundation for the formulation of an integrative service model encompassing BTC health prediction, diagnosis, intervention, prevention, and health augmentation.

### Study design

**Figure 1:**
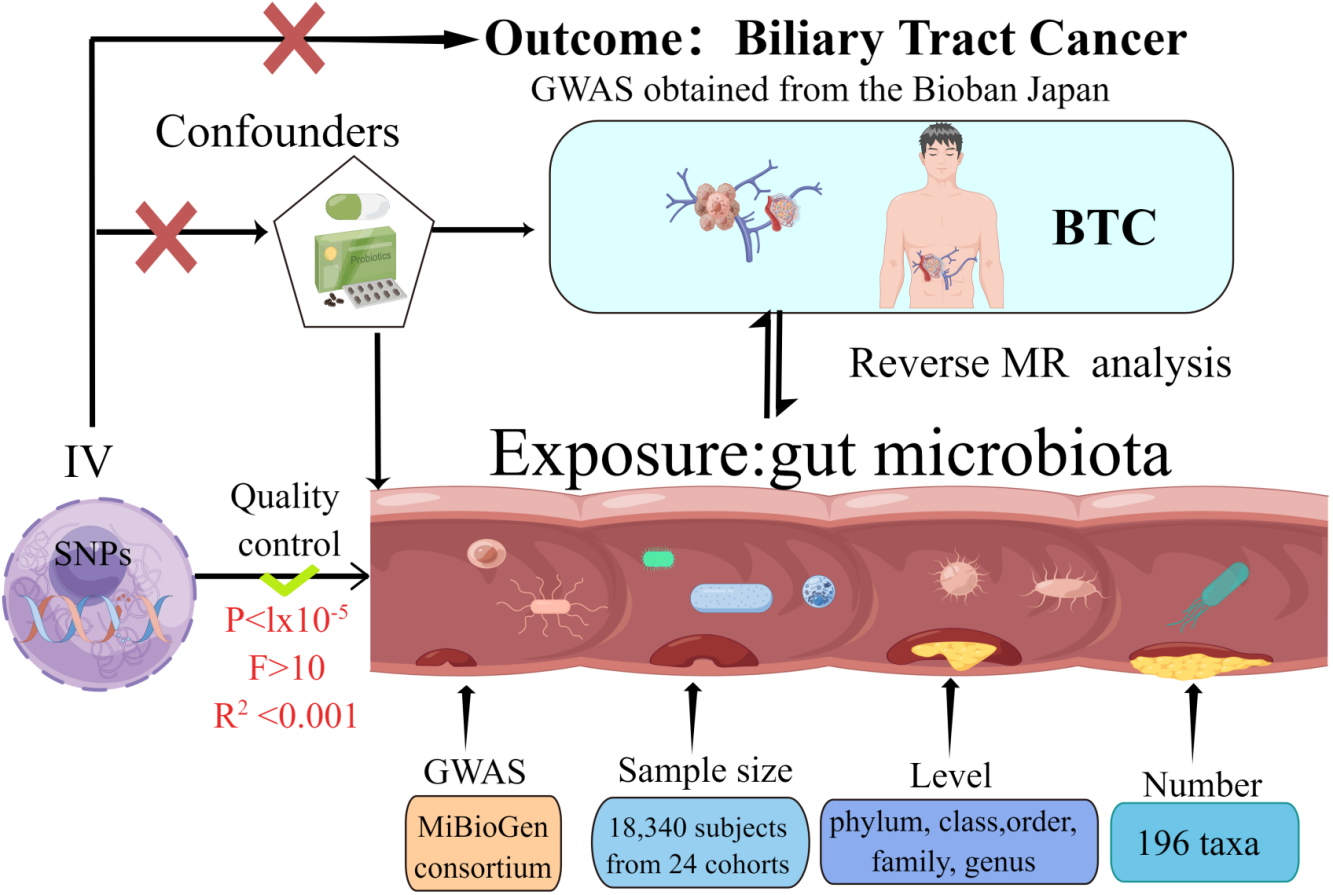
The study design of the present Mendelian randomization study of the associations of the gut microbiota and Biliary tract cancer risk.

The present MR investigation was conducted and documented in compliance with the STROBE-MR guidelines, which are designed to fortify the reporting quality of observational epidemiological studies (Skrivankova et al., 2021a; Skrivankova et al., 2021b). In order to evaluate our proposed hypothesis, we selected genetic variants pertinent to features of gut microbiota (N = 18,340) as well as BTC (comprising 418 cases and 159,201 controls) for our analysis. bidirectional MR approach was employed to scrutinize the influence of gut microbiota on the susceptibility to BTC development. MR integrates summary statistics from Genome-Wide Association Studies (GWAS), thereby attenuating the impact of confounding variables on the analysis. **Figure 1** delineates the schematic representation of the research methodology. The MR investigation adhered to three critical assumptions to ensure the validity and reliability of the resultant findings: (1) Instrumental variables (IVs) were distinctly correlated with each GM taxon and exhibited no association with BTC; (2) The IVs uniquely linked with each GM taxon demonstrated no correlation with potential confounding variables; and (3) The influence exerted by the IVs on BTC was mediated exclusively through their respective associations with each GM taxon, devoid of interference from extraneous variables.

## Materials

We sourced Single Nucleotide Polymorphisms (SNPs) correlated with gut microbial abundance from the GWAS conducted by the MiBioGen consortium. This study encompassed 25 cohorts comprising a total of 18,340 participants and was geared toward pinpointing genetic loci that exert an impact on the relative abundance of gut microbes. This identification was achieved through the analysis of 16S rRNA sequencing profiles corresponding to the study subjects. Subsequent to their collection, summary statistics from GWAS pertaining to 196 bacterial taxa were incorporated into the MR analysis (Kurilshikov et al., 2021). We retrieved GWAS summary statistics related to BTC from the BioBank Japan (BBJ). The BBJ serves as a prospective genomic repository that has collaboratively amassed DNA and serum specimens from a consortium of 12 medical institutions across Japan. Investigations pertaining to BBJ received ethical approval from both the Institutional Review Board at RIKEN Yokohama Institute and the Ethics Committee at the Institute of Medical Science, University of Tokyo (Ishigaki et al., 2020).

### Selection of IVs

A rigorous sequence of quality control measures was implemented to identify and select IVs that met the established eligibility criteria, In alignment with the prevailing standards for GM research, we selected IVs using a threshold of *P* < 1 × 10^−5^ for screening (Zhang et al., 2023). The F-statistic serves as a quantitative metric for assessing the robustness of the association between genetic variants and the exposure variable, with a higher F-statistic signifying a more potent instrumental variable. Only IVs that manifest an F-statistic exceeding 10 are retained for further analysis (Burgess et al., 2017). The calculation of linkage disequilibrium (LD) between SNPs was performed utilizing the European samples from the 1000 Genomes Project as the reference panel. SNPs manifesting the lowest *P*-values were retained for further study, provided they satisfied the criterion of R^2^ < 0.001 within a clumping window size of 10,000 kilobases. SNPs exhibiting a minor allele frequency (MAF) below the threshold of 0.01 were systematically excluded from the analysis.

### Statistical analysis

The MR investigation was integrated to assess the causative associations between 196 distinct microbial taxa and BTC. Utilizing the Bonferroni correction method, we delineated the criteria for statistical significance for the principal MR outcomes across various taxonomic levels, ranging from phylum to genus. Specifically, for a given taxonomic level that incorporates ‘n’ distinct bacterial taxa, the threshold for statistical significance, after applying Bonferroni correction, is determined as 0.05/n (Luo et al., 2023a). The inverse variance-weighted (IVW) methodology was employed as the principal analytical instrument for evaluating the influence of gut microbiota on the susceptibility to BTC (González-García et al., 2023).To enhance the robustness of our analytical approach, we incorporated four supplementary MR techniques into our investigation. These encompassed the simple mode, weighted mode, MR-Egger, and weighted median strategies. A *P*-value of less than 0.05 was considered to denote statistical significance in the context of MR analysis. In consideration of the limited sample size in the BTC GWAS, the statistical power for conducting a MR analysis was ascertained via computations performed on the mRnd website. (https://shiny.cnsgenomics.com/mRnd/) (Brion et al., 2013). Within the context of the IVW method, the Cochran’s Q test was employed to evaluate the extent of statistical heterogeneity among the SNPs incorporated in each individual analysis. The estimation of horizontal pleiotropy was conducted through the utilization of the MR-Egger intercept test, a *P*-intercept value of less than 0.05 serves as an indicative metric, suggesting the existence of horizontal pleiotropy. To mitigate the impact of potential confounding variables, we conducted an additional query using the PhenoScanner database to ascertain whether the SNPs that yielded significant MR estimates in this investigation were concomitantly linked with other risk factors for BTC. Furthermore, for those gut microbiota taxa exhibiting causal relationships, we conducted advanced pleiotropy evaluations utilizing the MR Pleiotropy RESidual Sum and Outlier (MR-PRESSO) methodology, subsequently excluding any identified outliers, Statistical evaluations were conducted utilizing the TwoSampleMR and MR-PRESSO packages within the R software environment (version 4.2.2).

### Reverse MR analysis

To examine the potential causal relationship between BTC and various bacterial genera, we conducted a reverse Mendelian Randomization analysis, wherein BTC served as the exposure variable and the composition of the gut microbiota was the outcome variable. SNPs linked to BTC were employed as instrumental variables for this analysis.

### Ethical approval

In prior research endeavors, written informed consents were duly obtained from all involved participants. Correspondingly, these studies received the necessary approvals from relevant ethical review committees (Kurilshikov et al., 2021; Ishigaki et al., 2020).

## Results

In the present study, initial efforts were made to obtain effective IVs using rigorous quality control. These IVs were then used in an MR analysis to assess the putative causal relationship between 196 GM taxa and BTC. In every retained SNP, the F-statistic exceeded the value of 10 (as detailed in **Supplementary Table**), signifying an adequate level of statistical power in the correlation between the IV and their corresponding bacterial taxa. For the entirety of the MR outcomes, we undertook sensitivity assessments to scrutinize both heterogeneity, as indicated by Cochran’s Q statistic, and pleiotropic effects, as evaluated through MR-Egger regression and MR-PRESSO methodologies.

### Causal effect of gut microbiota on BTC

**Figure 2:**
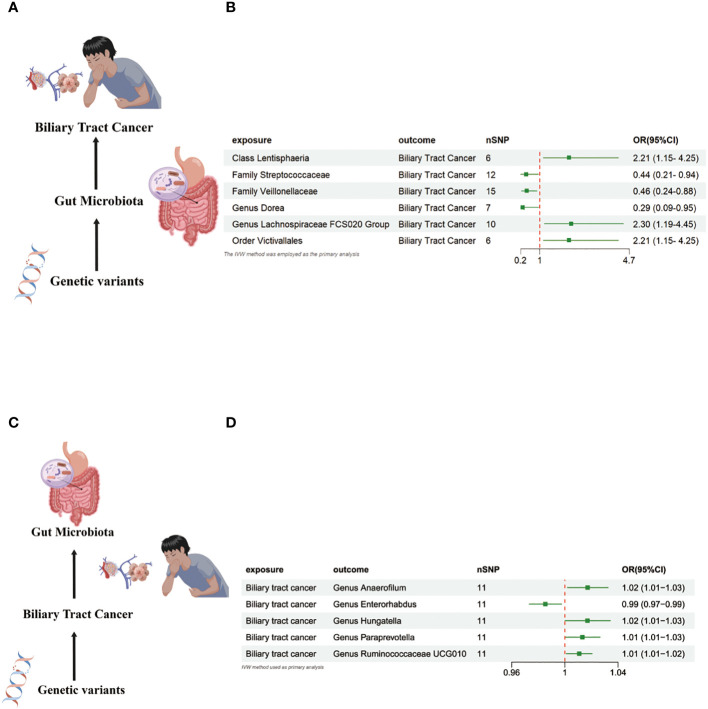
**(A)** Causal effect of gut microbiota with Biliary tract cancer Schematic representation of the MR analysis results. **(B)** Forest plot of the MR analysis results. **(C)** Causal effect of Biliary tract cancer with gut microbiota Schematic representation of the Reverse MR analysis results. **(D)** Forest plot of the MR analysis results. OR odds ratio, CI confidence interval, IVW inverse variance weighted method, Significant threshold was set at p-value <0.05 for the Inverse Variance Weighted method (IVW).

**Figure 3:**
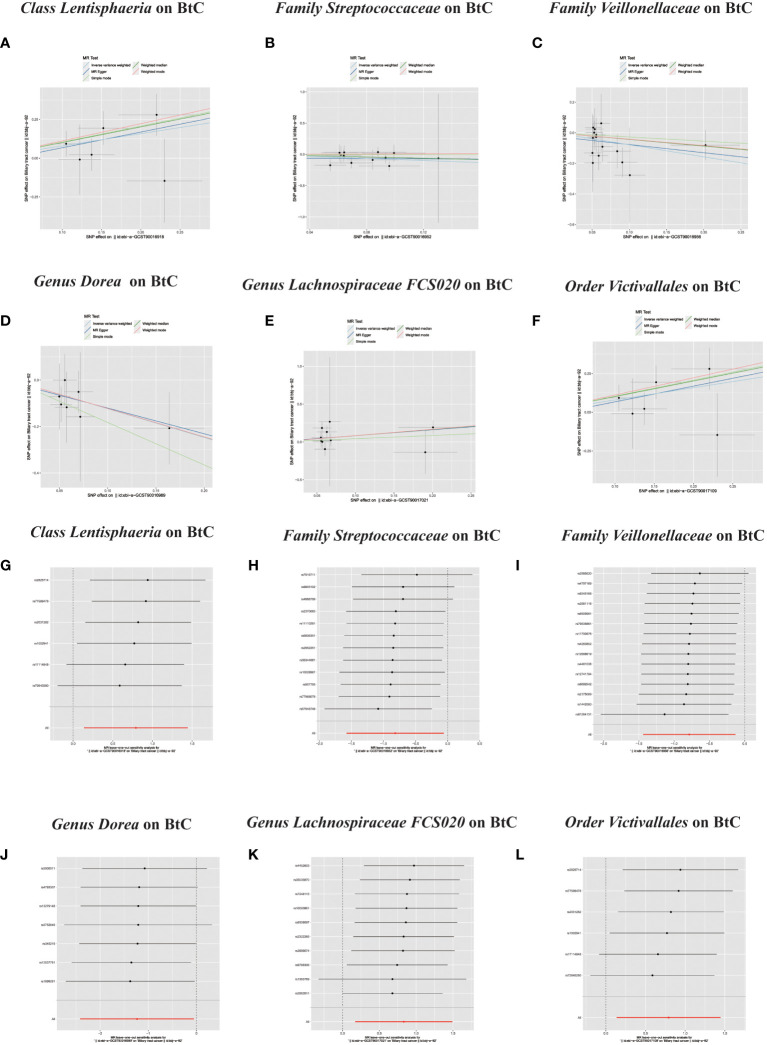
**(A–L)** Scatter plots of significant causality of the GM and BTC. Scatter plot of the effect size and 95% confidence interval of each SNP on GM and BTC risk. The horizontal axis reflects genetic effect of each SNP on GM. The vertical axis represents the genetic effect of each SNP on BTC risk. Leave-one-out analysis for the impact of individual SNPs on the association between GM and BTC risk. By leaving out exactly one SNP, it demonstrates how each individual SNP influences the overall estimate.

In the MR investigation conducted on gut microbiota, utilizing microbiota-associated SNPs as instruments, the initial analysis using the IVW method discerned six specific taxa that exhibited the potential for exerting causal influences on the occurrence of BTC. Utilizing the IVW analytical method, it was discerned that the *Family Streptococcaceae* (odds ratio (OR) 0.44, 95% confidence interval (CI) 0.21–0.94, *P* = 0.034); *Family Veillonellaceae* (OR 0.46, 95% CI 0.24–0.88, *P* = 0.018); *Genus Dorea* (OR 0.29, 95% CI 0.09–0.88, *P* = 0.018) exhibited a negative correlation with BTC susceptibility. Conversely, the *Class Lentisphaeria* (OR=2.21, 95% CI:1.15–4.25, *P*=0.017); *Genus Lachnospiraceae FCS020* (OR=2.30, 95% CI:1.19–4.45, *P*=0.013) and *Order Victivallales* (OR=2.21, 95% CI:1.15–4.25, *P*=0.017) were observed to have a positive association with BTC risk (**Figures 2A, B**, **3A–L**). The p-values derived from the Cochran Q test and the MR-Egger intercept test exceeded 0.05. This suggests compelling evidence supporting the lack of heterogeneity and the absence of pleiotropy in the study (**Table 1**).

**Table 1:** Summary results of MR (Target Gut microbiome on Biliary tract cancer).

Taxa	exposure	outcome	Nsnp	Methods	Beta	SE	OR (95%CI)	*P* value	MR-PRESSO	Heterogeneity		Horizontal pleiotrop		
										Cochran’s Q	*P* value	Egger intercept	SE	*P* value
Class	Lentisphaeria	Biliary tract cancer	6	MR-Egger	1.014	1.231	2.76 (0.25-30.82)	0.456	0.69	3.460	0.629	-0.034	0.182	0.859
				Weighted median	1.009	0.431	2.74 (1.18- 6.39)	0.019						
				Inverse variance weighted	0.791	0.334	2.21 (1.15- 4.25)	0.017						
				Simple mode	1.036	0.651	2.82 (0.79-10.11)	0.172						
				Weighted mode	1.112	0.509	3.04 (1.12- 8.26)	0.080						
Order	Victivallales	Biliary tract cancer	6	MR-Egger	1.014	1.231	2.76 (0.25-30.82)	0.456	0.68	3.460	0.629	-0.034	0.182	0.859
				Weighted median	1.009	0.442	2.74 (1.15- 6.53)	0.022						
				Inverse variance weighted	0.791	0.334	2.21 (1.15- 4.25)	0.017						
				Simple mode	1.036	0.581	2.82 (0.90- 8.82)	0.134						
				Weighted mode	1.112	0.530	3.04 (1.08- 8.60)	0.090						
Family	Streptococcaceae	Biliary tract cancer	12	MR-Egger	-0.120	1.955	0.89 (0.02-40.94)	0.952	0.62	8.212	0.694	-0.056	0.155	0.723
				Weighted median	-0.514	0.557	0.60 (0.20- 1.78)	0.355						
				Inverse variance weighted	-0.817	0.386	0.44 (0.21- 0.94)	0.034						
				Simple mode	-0.036	0.890	0.96 (0.17- 5.52)	0.968						
				Weighted mode	0.077	0.811	1.08 (0.22- 5.30)	0.925						
Family	Veillonellaceae	Biliary tract cancer	15	MR-Egger	-0.533	0.681	0.59 (0.15-2.23)	0.447	0.80	7.912	0.893	-0.023	0.055	0.677
				Weighted median	-0.435	0.474	0.65 (0.26-1.64)	0.358						
				Inverse variance weighted	-0.785	0.334	0.46 (0.24-0.88)	0.018						
				Simple mode	-0.272	0.783	0.76 (0.16-3.53)	0.732						
				Weighted mode	-0.419	0.453	0.66 (0.27-1.60)	0.370						
Genus	Dorea	Biliary tract cancer	7	MR-Egger	-1.105	1.473	0.33 (0.02-5.94)	0.486	0.98	0.971	0.986	-0.010	0.107	0.928
				Weighted median	-1.232	0.757	0.29 (0.07-1.29)	0.103						
				Inverse variance weighted	-1.231	0.603	0.29 (0.09-0.95)	0.041						
				Simple mode	-1.826	1.117	0.16 (0.02-1.44)	0.153						
				Weighted mode	-1.221	0.855	0.29 (0.06-1.58)	0.203						
Genus	Eubacterium hallii Group	Biliary tract cancer	11	MR-Egger	-0.062	0.665	0.94 (0.26- 3.46)	0.927	0.64	7.393	0.687	0.100	0.062	0.145
				Weighted median	0.622	0.487	1.86 (0.72- 4.85)	0.201						
				Inverse variance weighted	0.838	0.351	2.31 (1.16- 4.61)	0.017						
				Simple mode	1.198	0.716	3.31 (0.81-13.49)	0.125						
				Weighted mode	0.619	0.446	1.86 (0.77- 4.46)	0.195						
Genus	Lachnospiraceae FCS020 Group	Biliary tract cancer	10	MR-Egger	0.774	0.682	2.17 (0.57-8.27)	0.289	0.58	8.596	0.475	0.005	0.060	0.924
				Weighted median	0.820	0.519	2.27 (0.82-6.29)	0.114						
				Inverse variance weighted	0.832	0.337	2.30 (1.19-4.45)	0.013						
				Simple mode	0.426	0.758	1.53 (0.35-6.78)	0.588						
				Weighted mode	0.840	0.454	2.32 (0.95-5.65)	0.097						

### Causal effect of BTC on gut microbiota

**Figure 4:**
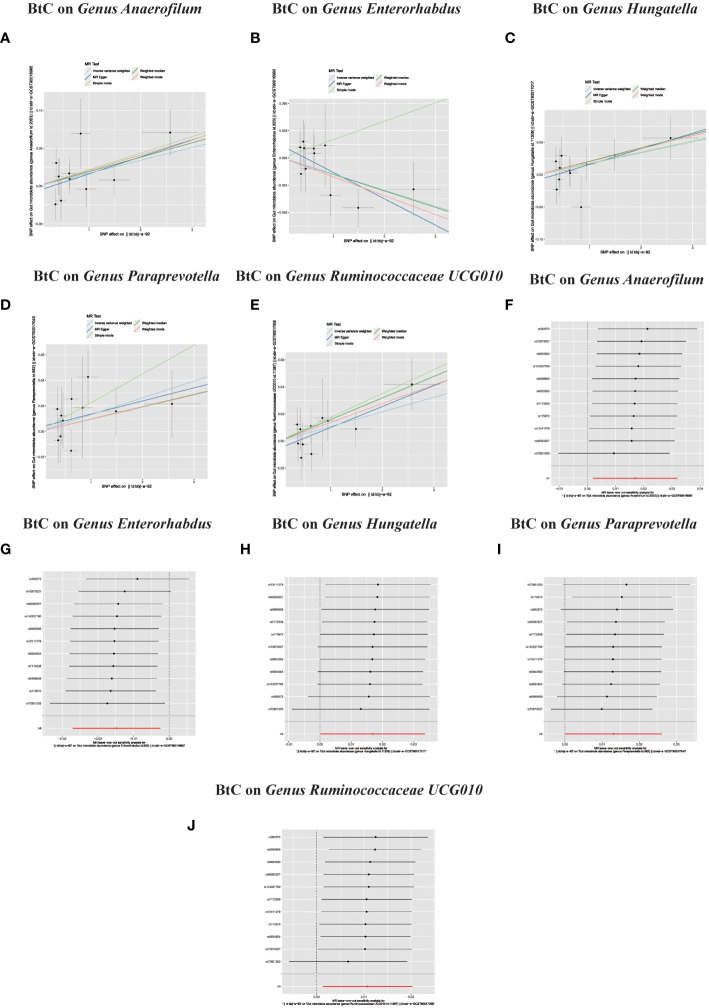
**(A–J)** In reverse MR analysis, Plots for “leave-one-out” analysis for causal effect of Biliary tract cancer on gut microbiota risk; In reverse MR analysis, The scatter plots for association between Biliary tract cancer and gut microbiota.

In the bidirectional MR study, we examined the potential causal linkage between BTC on gut microbiota. Our analysis revealed a marked causal relationship between genetically inferred BTC and an elevated abundance of the following genera: *Anaerofilum* (OR = 1.02, 95% CI: 1.01–1.03, *P* = 0.026), *Hungatella* (OR = 1.02, 95% CI: 1.01–1.03, *P* = 0.026), *Paraprevotella* (OR = 1.02, 95% CI: 1.01–1.03, *P* = 0.026), and *Ruminococcaceae UCG010* (OR = 1.02, 95% CI: 1.01–1.03, *P* = 0.026). Conversely, a reduced abundance was observed in the *Genus Enterorhabdus* (OR = 0.99, 95% CI: 0.97–0.99, P = 0.018) (**Figures 2C, D**, **4A–J**). The consistency of the outcomes was further assessed utilizing the MR Egger approach. The obtained p-values exceeding 0.05 suggest a lack of heterogeneity in our findings. Subsequent evaluation using the MR-PRESSO methodology indicated the absence of significant outliers and a lack of horizontal pleiotropy within our MR study (P > 0.05) (**Table 2**).

**Table 2:** Summary results of bidirectional MR (Biliary tract cancer on target Gut microbiome).

Taxa	exposure	outcome	Nsnp	Methods	Beta	SE	OR (95%CI)	*P* value	MR-PRESSO	Heterogeneity		Horizontal pleiotrop		
										Cochran’s Q	*P* value	Egger intercept	SE	*P* value
Genus	Biliary tract cancer	Anaerofilum	11	MR-Egger	0.022	0.012	1.02 (1.00-1.05)	0.114	0.578	8.280	0.601	-0.006	0.011	0.608
				Weighted median	0.019	0.011	1.02 (1.00-1.04)	0.086						
				Inverse variance weighted	0.016	0.007	1.02 (1.01-1.03)	0.026						
				Simple mode	0.021	0.015	0.97 (0.91-1.03)	0.179						
				Weighted mode	0.020	0.010	1.02 (1.00-1.04)	0.077						
Genus	Biliary tract cancer	Enterorhabdus	11	MR-Egger	-0.024	0.010	0.98 (0.96-1.00)	0.047	0.652	7.957	0.632	0.010	0.009	0.293
				Weighted median	-0.015	0.009	0.99 (0.97-1.00)	0.106						
				Inverse variance weighted	-0.014	0.006	0.99 (0.97-0.99)	0.018						
				Simple mode	0.016	0.015	1.02 (0.99-1.05)	0.317						
				Weighted mode	-0.017	0.008	0.98 (0.97-1.00)	0.075						
Genus	Biliary tract cancer	Hungatella	11	MR-Egger	0.024	0.014	1.02 (1.00-1.05)	0.123	0.934	4.998	0.891	-0.008	0.013	0.538
				Weighted median	0.020	0.011	1.02 (1.00-1.04)	0.064						
				Inverse variance weighted	0.016	0.008	1.02 (1.01-1.03)	0.047						
				Simple mode	0.017	0.015	1.02 (0.99-1.05)	0.287						
				Weighted mode	0.020	0.010	1.02 (1.00-1.04)	0.082						
Genus	Biliary tract cancer	Paraprevotella	11	MR-Egger	0.010	0.011	1.01 (0.99-1.03)	0.378	0.814	6.556	0.766	0.003	0.010	0.757
				Weighted median	0.009	0.008	1.01 (0.99-1.03)	0.281						
				Inverse variance weighted	0.013	0.006	1.01 (1.01-1.03)	0.048						
				Simple mode	0.022	0.015	1.02 (0.99-1.05)	0.165						
				Weighted mode	0.009	0.008	1.01 (0.99-1.03)	0.282						
Genus	Biliary tract cancer	Ruminococcaceae UCG010	11	MR-Egger	0.015	0.008	1.02 (1.00-1.03)	0.088	0.912	4.703	0.910	-0.005	0.007	0.486
				Weighted median	0.015	0.006	1.02 (1.00-1.03)	0.020						
				Inverse variance weighted	0.010	0.004	1.01 (1.00-1.02)	0.026						
				Simple mode	0.017	0.009	1.02 (1.00-1.04)	0.106						
				Weighted mode	0.013	0.006	1.01 (1.00-1.03)	0.051						

## Discussion

The study at hand validates the initial hypothesis. The adopted methodology underscores a paradigmatic transition from reactive healthcare to a framework emphasizing predictive, preventive, and personalized medicine (3PM/PPPM), targeting susceptible segments of the populace. The worldwide prevalence of BTC has consistently presented a considerable challenge to public health initiatives over an extended period (Zhang Y. et al., 2021). Biobanking is transitioning into a new epoch characterized by the prominence of big data, in the age of big data, biobanking plays a pivotal role in advancing predictive, preventive, and personalized medicine, ensuring optimal treatment for each patient at the appropriate juncture. In this research, we harnessed expansive GWAS summary-level data through MR analysis to scrutinize the putative causal linkage between the gut microbiota and BTC. The results indicate that different constituents of the gut microbiota might either augment or attenuate BTC risk. In the context of big data, biobanking facilitates the transition towards a more individualized healthcare framework. PPPM offers an innovative methodology for addressing BTC-related disorders.The current research revealed that taxa from the *Family Streptococcaceae, Family Veillonellaceae*, and the *Genus Dorea* exhibited a negative correlation with BTC susceptibility. Conversely, taxa from the *Class Lentisphaeria, Order Victivallales*, and the *Genus Lachnospiraceae FCS020 Group* demonstrated a positive association with BTC risk.

The *Streptococcaceae family*, particularly some strains of *Streptococcus*, have been investigated for their potential role in carcinogenesis (Re et al., 2023). One hypothesis is that they may induce chronic inflammation, a known risk factor for cancer. Chronic inflammation can lead to DNA damage, promoting mutations and the initiation of cancerous growths. Furthermore, certain Streptococcus species produce metabolites that may have carcinogenic properties, potentially impacting the development of BTC (Plieskatt et al., 2013; Daniel et al., 2023). Members of the *Veillonellaceae family* are known for their role in the fermentation of proteins and carbohydrates in the gut. The metabolites produced during this process, such as short-chain fatty acids, can have both protective and harmful effects. While some metabolites might have anti-inflammatory and anti-carcinogenic effects, others may contribute to the development of cancer. For BTC, the pathogenic mechanism may involve the alteration of bile acid metabolism and the gut-liver axis, which can lead to an imbalance in the liver’s cellular environment, potentially contributing to carcinogenesis (Mao et al., 2021; Button et al., 2023). *Dorea*, a less studied genus, has been linked with various gastrointestinal diseases. Its role in BTC could be associated with the modulation of the gut-liver axis and immune responses. Dorea may influence the liver’s immune environment, either promoting or inhibiting inflammation. In the context of BTC, an altered immune response in the liver could facilitate the development of cancerous cells or, conversely, provide a protective effect (Mima et al., 2017).

In the bidirectional MR study, we examined the potential causal linkage between BTC on gut microbiota. The gastrointestinal tract harbors a diverse and intricate consortium of microorganisms, collectively referred to as the gut microbiota. The portal vein conveys metabolites originating from the gut to the liver. Molecules such as bile acids, which are secreted by the liver, modulate the microbial environment. Binda et al. delineated three primary modalities through which gut microbiota potentially instigate cancerous activities: firstly, through bacterial toxins and metabolites; secondly, by altering both local and systemic immune responses of the host; and thirdly, via metabolic alterations in both the microbiota and the host (Binda et al., 2022). To a certain degree, our findings concur with earlier research (Mao et al., 2021). In clinical investigations analyzing stool samples from BTC patients, there was a notable increase in alpha-diversity compared to healthy counterparts. Specifically, the abundance of taxa such as *Lactobacillus, Actinomyces, Peptostreptococcaceae, Alloscardovia*, and *Bifidobacteriaceae* was conspicuously elevated (Jia et al., 2020). Nevertheless, the integration of such genetic information into clinical protocols presents a formidable challenge. Our research effectively established a connection between the gut microbiota and BTC by employing MR, considering genetic data as IV, and extrapolating the genetic association between the two entities. This represents the inaugural MR study elucidating the causal relationship between the gut microbiota and BTC, effectively mitigating the influence of confounders. Furthermore, the outcomes from the MR study hold significant relevance for public health. They augment prior research concerning the gut microbiota and BTC, offering a novel viewpoint on their genetic-level association. In terms of disease prevention, timely modulation of the gut microbiota can steer the prophylaxis of BTC disease. Diagnostically, it’s imperative to prioritize BTC screenings for individuals exhibiting gut microbiota disturbances. This research utilized GWAS data on intestinal flora from European subjects and BTC GWAS data from Japanese subjects. Consequently, the population sample in this investigation offers a representative cross-section. It is imperative to acknowledge the constraints inherent to our research. First, the genetic information pertaining to BTC was sourced from GWAS. However, the relatively small sample size of cancer cases could potentially skew the results of the GWAS, given the imbalanced ratios between cases and controls; Second, our projections may also be susceptible to inherent limitations associated with Mendelian Randomization analysis, including potential selection bias.

From a broader perspective, these bacteria and their interactions with the host’s immune system, as well as their metabolic by-products, are of significant interest in understanding BTC. The gut microbiota can modulate systemic inflammation, immune surveillance, and the metabolome, all of which are crucial factors in carcinogenesis. It is essential to note that while these associations are promising, they are complex and require further research. Future studies should focus on elucidating the precise mechanisms by which these bacteria influence BTC, potentially leading to new therapeutic strategies. The rapidly evolving field of microbiome research continually provides new insights into the intricate relationship between the gut flora and various cancers, including BTC.

## Conclusion

Our inaugural systematic Mendelian randomization assessment furnishes empirical support suggesting a potential causal linkage between various gut microbiota taxa and BTC. This finding could offer salient biomarkers that are advantageous for the early, non-invasive detection of BTC. Furthermore, it might delineate prospective targets for therapeutic strategies in addressing the ailment. While extensive research is still needed to elucidate the connections between the gut microbiome and BTC, burgeoning mechanistic understandings are paving the way for innovative interventions, including potential strategies for microbiota modulation. Furthermore, these insights are shaping public health recommendations concerning dietary and lifestyle habits to preemptively combat these fatal malignancies. Additional scholarly investigations are imperative to both authenticate particular gut microbes linked to BTC and to substantiate the underlying mechanism and causal relationship through animal models and clinical studies. Ultimately, this progression will facilitate the shift from reactive medical interventions to a PPPM approach in the oversight of BTC.

## Data availability statement

The datasets presented in this study can be found in online repositories. The names of the repository/repositories and accession number(s) can be found in the article/**Supplementary Material**.

## Ethics statement

The studies involving humans were approved by All the data utilized in this investigation are publicly accessible and fall within the public domain. All participants granted informed consent, and the study protocols received approval from their respective local ethical committees. The studies were conducted in accordance with the local legislation and institutional requirements. The participants provided their written informed consent to participate in this study. Written informed consent was obtained from the individual(s) for the publication of any potentially identifiable images or data included in this article. Consensus among all authors was achieved regarding the manuscript.

## Author contributions

KW: Conceptualization, Data curation, Formal analysis, Investigation, Methodology, Software, Visualization, Writing – original draft, Writing – review & editing, Supervision. SW: Writing – original draft, Writing – review & editing, Data curation, Methodology, Supervision, Conceptualization, Formal analysis, Validation, Investigation, Visualization, Software. XQ: Conceptualization, Investigation, Methodology, Software, Writing – original draft, Writing – review & editing, Project administration, Supervision, Validation, Data curation. YFC: Conceptualization, Investigation, Methodology, Software, Writing – original draft, Writing – review & editing, Project administration, Validation, Supervision. YHC: Writing – review & editing, Data curation, Methodology, Supervision, Conceptualization, Validation, Investigation, Software. JW: Conceptualization, Data curation, Formal analysis, Investigation, Project administration, Software, Writing – original draft. YZ: Investigation, Methodology, Project administration, Software, Supervision, Validation, Writing – review & editing. QG: Conceptualization, Data curation, Investigation, Methodology, Project administration, Supervision, Writing – review & editing. CZ: Conceptualization, Data curation, Funding acquisition, Investigation, Methodology, Project administration, Resources, Supervision, Writing – review & editing. DZ: Conceptualization, Data curation, Formal analysis, Funding acquisition, Investigation, Methodology, Project administration, Resources, Supervision, Writing – review & editing.

## Supplementary material

Supplementary Table 1 Instrumental variables (selected SNPs) used in MR analysis of the association between gut microbiota and Biliary tract cancer.

Supplementary Table 2 Instrumental variables (selected SNPs) used in bidirectional MR analysis of the association between Biliary tract cancer and gut microbiota.
